# Region-Dependent Modulation of Neural Plasticity in Limbic Structures Early after Traumatic Brain Injury

**DOI:** 10.1089/neur.2020.0045

**Published:** 2021-04-08

**Authors:** Ann N. Hoffman, Sonya Watson, Michael S. Fanselow, David A. Hovda, Christopher Giza

**Affiliations:** ^1^ Department of Neurosurgery, Brain Injury Research Center, University of California, Los Angeles, Los Angeles, California, USA.; ^2^ Department of Psychology, University of California, Los Angeles, Los Angeles, California, USA.; ^3^ Staglin Center for Brain and Behavioral Health, University of California, Los Angeles, Los Angeles, California, USA.; ^4^ Department of Psychiatry and Biobehavioral Sciences, University of California, Los Angeles, Los Angeles, California, USA.; ^5^ Steve Tisch BrainSPORT Program, University of California, Los Angeles, Los Angeles, California, USA.; ^6^ Department of Medical and Molecular Pharmacology, University of California, Los Angeles, Los Angeles, California, USA.; ^7^ Mattel Children's Hospital, University of California, Los Angeles, Los Angeles, California, USA.

**Keywords:** adult brain injury, animal studies, neuroplasticity, receptors

## Abstract

Traumatic brain injury (TBI)-induced disruptions in synaptic function within brain regions and across networks in the limbic system may underlie a vulnerability for maladaptive plasticity and contribute to behavioral comorbidities. In this study we measured how synaptic proteins respond to lateral fluid percussion injury (FPI) brain regions known to regulate emotion and memory, including the basolateral amygdala (BLA), dorsal and ventral hippocampus (DH, VH), and medial prefrontal cortex (PFC). We investigated proteins involved in regulating plasticity, including synaptic glutamatergic a-amino-3-hydroxy5-methyl-4-isoxazolepropionic acid (AMPA; GluA1, GluA2) and *N-*methyl-D-aspartate (NMDA; NR1, NR2A, NR2B) receptor subunits as well as inhibitory gamma-aminobutyric acid (GABA) synthetic enzymes (GAD67, GAD65) via western blot. Adult male rats received a mild-moderate lateral FPI or sham surgery and ipsi- and contralateral BLA, DH, VH, and PFC were collected 6 h, 24 h, 48 h, and 7 days post-injury. In the ipsilateral BLA, there was a significant decrease in NR1 and GluA2 24 h after injury, whereas NR2A and NR2B were increased in the contralateral BLA at 48 h compared with sham. GAD67 was increased ipsilaterally at 24 h, but decreased contralaterally at 48 h in the BLA. In the DH, both NMDA (NR2A, NR2B) and GABA-synthetic (GAD65, GAD67) proteins were increased acutely at 6 h compared with sham. GAD67 was also robustly increased in the ipsilateral VH at 6 h. In the contralateral VH, NR2A significantly increased between 6 h and 24 h after FPI, whereas GAD65 was decreased across the same time-points in the contralateral VH. In the medial PFC at 24 h we saw bilateral increases in GAD67 and a contralateral decrease in GluA1. Later, there was a significant decrease in GAD67 in contralateral PFC from 48 h to 7 days post-injury. Collectively, these data suggest that lateral FPI causes a dynamic homeostatic response across limbic networks, leading to an imbalance of the proteins involved in plasticity in neural systems underlying cognitive and emotional regulation.

## Introduction

Traumatic brain injury (TBI) affects an estimated 2.8 million people every year in the United States,^1^ and is defined as a disruption of the normal function of the brain caused by an external biomechanical force or a penetrating head injury.^2,3^ Although estimates vary, 75–90% of TBIs are classified as mild in which there is little to no loss of consciousness^4,5^; however, psychiatric comorbidities have been reported after mild to severe TBI.^6–8^ Focal and diffuse damage to tissue can result in a disruption of functional circuits, including alterations to neuroplasticity mechanisms.^9^

Neuroplasticity at the cellular level can involve changes in the excitability of a single neuron through alterations in the availability of glutamate receptors that ultimately can alter the rate of signal propagation between cells. Glutamatergic receptors including a-amino-3-hydroxy5-methyl-4-isoxazolepropionic acid (AMPA) and *N*-methyl-D-aspartate (NMDA) receptors allow for propagation of cell signaling. AMPA receptors (AMPArs) allow sodium and potassium into the cell when activated by glutamate, whereas NMDA receptors (NMDArs) can bind multiple agonists including glutamate, resulting in the influx of calcium, potassium, and sodium. NMDArs are comparatively more selective in their activation due to the magnesium ion channel block, which is only removed under certain excitatory conditions, making NMDArs more modulatory in excitatory transmission and AMPArs primarily responsible for fast transmission.

Complementary to glutamatergic processes are gamma-aminobutyric acid (GABA)ergic processes that act to inhibit neuronal excitability by allowing negative ions to flow into the cell, causing hyperpolarization and consequently decreasing the potential for firing. Glutamate decarboxylase (GAD) enzymes are produced and serve to convert glutamate to GABA for inhibitory transmission. Together, glutamatergic and GABAergic processes serve as prime regulators of neuronal activity by adjusting the rate and potential of ionic flow within and across neurons. Importantly, these mechanisms, which enable rewiring of the brain, have been found to be disrupted in various brain regions following TBI.^10–14^ Alterations of neural plasticity mechanisms within and across neural networks after injury could give rise to symptoms commonly observed after brain injury, such as cognitive impairments and behavioral disturbances.

Clinical studies have reported high rates of psychiatric and mood-related comorbidities following TBI.^8,15–18^ To better understand this high comorbidity rate, it is important to consider how TBI may be affecting plasticity in brain regions such as the limbic system, which is important for mood and emotion. The limbic system comprises cortical and subcortical structures that work in unison to support a variety of functions including emotion, behavioral regulation, and long-term memory. One such region of the limbic system that may be altered after TBI is the amygdala. The amygdala is known to play a role in fear, anxiety, learning, and memory and is also highly implicated in psychiatric conditions such as anxiety and post-traumatic stress disorder (PTSD).^19^

Pre-clinical work supports a role in which the amygdala has increased plasticity following TBI,^12,20–22^ which may underlie the post-injury enhancement of fear and defense reactions. Additional limbic candidates underlying behavioral alterations following TBI include both the dorsal and ventral hippocampus as well as the prefrontal cortex. The dorsal hippocampus (DH) primarily performs cognitive functions and is pertinent to spatial and contextual memory, whereas the ventral hippocampus (VH) has a more prominent role in emotion, stress, and anxiety,^23–27^ both being vulnerable to disruptions by TBI. Lastly, the prefrontal cortex (PFC) is involved in the modulation of these subcortical networks in aversive stimuli processing^26,28–31^ and is therefore also a proximal anatomical candidate for functional changes in mood and psychiatric conditions after TBI.

The current study aimed to characterize the state of proteins that support the molecular excitatory/inhibitory balance of these limbic regions early after lateral fluid percussion injury (FPI). We investigated left lateral FPI induced changes of synaptic glutamatergic AMPA and NMDA receptor subunits as well as GABAergic GAD proteins in the amygdala-hippocampal-prefrontal limbic network at multiple time-points within the first week after injury. We hypothesized that TBI would lead to differential expression of proteins that support synaptic excitatory and inhibitory processes in these limbic structures and PFC that may give rise to maladaptive emotional processing. Considering the high prevalence of psychiatric comorbidities in clinical populations with TBI, particularly the increased prevalence of anxiety and PTSD, we predicted that the amygdala would exhibit increased expression of proteins that support excitatory processes and/or reduced inhibitory-related protein expression. Further, given the functional connectivity of the structures examined and their coordinated role in emotion and cognition, we predicted dynamic alterations across regions and proteins within the first week following injury. The current study provides an initial investigation and overall picture of the regional dynamic molecular cascade in proteins that support synaptic excitatory and GABA synthesis across limbic structures within subjects in the subacute window after lateral TBI.

## Methods

### Subjects

Young adult male Sprague-Dawley rats (*n* = 45; Envigo; 250–275 g/8–9 weeks old upon arrival, 9–10 weeks old at time of injury or sham surgery) were pair housed on a regular 12:12 light cycle and received food and water *ad libitum*. Young adult males were utilized due to epidemiological data supporting males as being at a significantly higher risk for TBI, with the highest male-to-female ratios occurring in young adulthood.^32^ Ongoing and future experiments aim to investigate sex differences across these variables. Animals were handled daily (1 min/day) for 4 days prior to surgery, as is standard practice in our laboratory, to familiarize them to the experimenters and reduce stress-induced variability in our data. All procedures in this experiment were in accordance with the Institutional Animal Care and Use Committee at University of California, Los Angeles (UCLA). Rats were randomly assigned to either lateral FPI or sham surgery, and tissue was collected at either 6 h, 24 h, 48 h, or 7 days post-surgery. These time-points were chosen to capture times within a subacute window following impact in which we found the injured brain is undergoing metabolic recovery and dynamic alterations in plasticity mechanisms^9,11,33,34^ and may be vulnerable to stressor exposure based on our behavior experiments.^12,35^ Within a surgery cohort, injury severity for FPI animals collected across time-points was balanced according to latency to toe pinch withdrawal (see Table 1).

**Table 1.** Balanced Injury Severity across Experimental Time-Points

Group	Apnea (sec)	Toe pinch withdrawal (sec)	Atmospheres of pressure
6 h	24.5 ± 23.5	305.1 ± 230.8	3.2 ± 0.4
24 h	20.1 ± 17.5	249.6 ± 161.9	2.7 ± 0.2
48 h	15.0 ± 12.4	285.8 ± 78.9	2.7 ± 0.1
7 days	33.4 ± 33.8	358.5 ± 161.5	2.9 ± 0.3

### Lateral fluid percussion injury

**FIG. 1.**
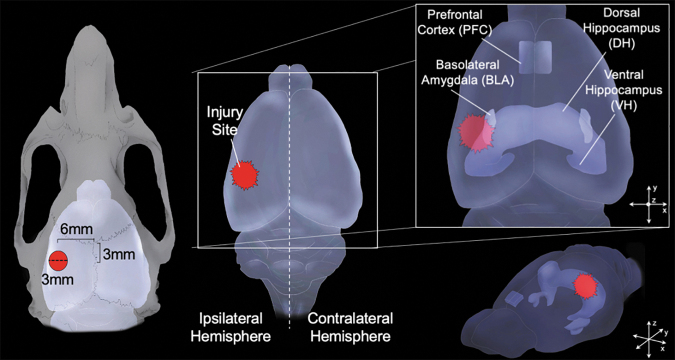
Targeted anatomy. (Left) Diagram of rat skull indicates location of craniectomy (Center). Model brain indicates positioning of diffuse injury caused by lateral fluid percussion injury (FPI). Right inset demonstrates regions of interest; basolateral amygdala (BLA), dorsal and ventral hippocampus (DH, VH, respectively), and medial prefrontal cortex (PFC).

Lateral FPI was induced using a previously published protocol^9,11,12,36^ typically used in our laboratory. Briefly, animals were anesthetized under a 1–2% isoflurane-oxygen mixture and maintained at ∼3%. A midline incision was made followed by a left hemisphere 3-mm diameter craniectomy centered 3 mm posterior and 6 mm lateral to bregma as illustrated in Figure 1. A plastic injury cap was adhered to the skull with silicone gel and dental cement and filled with sterile saline. The animal was removed from anesthesia and the injury cap was attached to the FPI device (Virginia Commonwealth University, Richmond, VA). Upon eliciting a positive toe pinch response, a brief fluid pulse (∼20 msec) of saline was administered directly to the dura mater. Apnea and loss of consciousness (LOC; measured by return of toe pinch response) were measured, and rats were placed back on anesthesia to remove the injury cap and suture the scalp. Sham animals received the same surgical procedures but did not receive impact. Upon completion of surgery, animals were placed in a heated recovery chamber until normal behavior resumed, and they were subsequently returned to their home cage.

### Tissue collection

Following FPI or sham surgery, animals were pseudorandomized into four different groups for tissue collection time-points (6 h, 24 h, 48 h, and 7 days) to determine the dynamic time course in changes in proteins that support excitatory and inhibitory (GABA synthesizing) plasticity-related proteins in various regions of the corticolimbic network. At the appropriate time after injury for each animal, rats were deeply anesthetized with isoflurane, and brains were removed and immediately microdissected and frozen on dry ice. The ipsi- and contralateral medial PFC, DH, VH, and basolateral amygdala (BLA) were collected and stored at −80°C for analysis (see Fig. 1). Tissue samples were prepared using the Syn-PER Reagent protocol (Thermo Fisher) for enriched synaptic fractions (SNS) and crude homogenate (CH) to be analyzed separately. Briefly, Syn-PER Reagent was added to tissue samples and homogenized with a pestle homogenizer. All samples were then spun in a refrigerated 4°C centrifuge at 1200*g* for 10 min to rid the sample of large cellular debris via pellet formation. From this sample, a portion of the supernatant was then collected for CH protein and stored at 80°C. The remaining portion of the supernatant was spun at 15,000*g* for 20 min at 4°C to enhance the synaptic protein ratio in the samples, and pellets were resuspended in Syn-Per Buffer. All samples were stored at −80°C for further analysis via immunoblotting.

### Immunoblot

Western blots were performed using a standard protocol used in our laboratory.^11,13,37^ A series of pilot experiments were conducted to optimize protein amount loaded and antibody primary and secondary concentrations, specific to each brain region. The parameters outlined below were used for experimental tissue. The total amount of protein in each SNS and CH sample was determined with a Pierce BCA Protein Assay Kit. Protein was pseudo-randomly loaded to balance experimental conditions across each gel at a concentration of 0.33mg/mL on a 10% Tris-HCl gel and was run at 160 V for 50 min. To compare across gels, the same sham samples were repeated across blots within each regional analysis. Due to limitation of lanes (*n* = 24), blots were prepared with animals from sham, 6 h, and 24 h groups or sham, 48 h, and 7 day groups (*n* = 6–8 per group). The proteins were transferred from the gel to a nitrocellulose membrane at 0.4 A for 120 min. To image for total protein, the membrane was stained with SYPRO™ Ruby Protein Stain solution and imaged with a Bio-Rad imager with filter. The membrane was then blocked for 60 min in 5% milk in Tris-buffer saline with Tween 20 (TBST).

Primary antibodies were added and incubated overnight at 4°C for at least 16 h, in concentrations as follows; NR1 1:1000 (Millipore Sigma, AB9864), Sigma 1 1:500 (Thermo Fisher, 42-3300), NR2B 1:1000 (Millipore Sigma, AB1557P), NR2A 1:1000 (Millipore Sigma, AB1555P), GluR1 1:5000 (Abcam, AB31232), GluR2 1:5000 (Millipore Sigma, MABN1189). The CH immunoblots were probed for glutamate decarboxylase (GABA-synthetic) enzymes GAD67 1:1000 (Chemicon, MAB5406) then stripped, blocked, and probed for GAD65 1:5000 (Thermo Fisher, PA5-21297). Despite their close proximity in kDa weight, later tests with fluorescent immunoblotting demonstrated clear separation of these proteins. The blots were incubated with Goat-Anti Rabbit (Thermo Fisher, 65-6120) or Goat-Anti mouse (Thermo Fisher, 62-6700) secondary antibodies with a 1:5000 (Gad67, NR2B) or 1:10,000 (NR1, GAD65, NR2A, GluR1, GluR2) dilution in 1% milk TBST. Protein bands were developed using Bio-Rad ECL and were imaged on a Bio-Rad imager and analyzed using Bio-Rad Image Lab software. Raw receptor subunit pixel density was normalized to total protein pixel density within each lane. These normalized protein values from each sample were represented as percent change from the average of each sham group within a blot. The density of glutamatergic proteins (NMDAr and AMPAr subunits) is greatest at the synapse; therefore, we measured the excitatory-supporting post-synaptic proteins within SNS to enhance the signal and reduce variability in the sample. GAD proteins were measured to investigate the state of inhibitory tone across time after FPI across the selected regions of interest. GAD67 is present throughout the cell, whereas GAD65 is localized within the synaptic elements^38^; therefore, we chose to measure both enzymatic GAD proteins in the CH that would allow for the whole cell, and non-synaptic elements to allow for consistency across both proteins.

### Western blot analysis

Imaged blots were analyzed using Bio-Rad Image Lab software. Raw receptor subunit pixel density was normalized to total protein pixel density within each lane. These normalized protein values from each sample were represented as percent change from the average of each sham group within a blot. For statistical analyses, the ratio of receptor volume to total protein volume was compared across groups. See Supplementary Figure S1 for representative blots in detected significant effects.

### Statistical analysis

Given the invasive conditions for our sham surgery procedure and its implications, a pilot experiment was conducted to determine if there were potential changes in molecular signatures over time in response to sham surgery alone. Stressors caused by surgery and isoflurane exposure were most likely to affect the amygdala, thus tissue from the contralateral BLA of sham animals was assessed for each time-point, (∼*n* = 3/time-point). No significant differences were observed in the proteins investigated across post-surgery time-points (GluA1: F[3,6] = 0.53, *p =* 0.68; GluA2: F[3,6] = 0.83, *p =* 0.52; NR1: F[3,6] = 1.37, *p =* 0.34; NR2A: F[3,6] = 1.78, *p =* 0.25; NR2B: F[3,6] = 0.38, *p =* 0.77; GAD65: F[3,6] = 0.83, *p =* 0.52; GAD67: F[3,6] = 0.81, *p =* 0.53). Therefore, data from the sham conditions were combined into one sham group, which was compared with each of the post-FPI time-points.

Due to large sample size and limited number of wells/gel, data were analyzed according to the following methods; for each brain region and each hemisphere subjects were pseudo-randomized across blots so that the collective sham group (*n* = 8) was represented on every blot with two of the FPI groups (i.e., Blot 1: sham, 6 h [*n* = 8] and 24 h [*n* = 7]; Blot 2: replicate sham, 48 h [*n* = 6] and 7 days [*n* = 8]). Data were analyzed by one-way analysis of variance (ANOVA) within blot to reveal FPI group change relative to sham. Comparisons between early (6 h vs. 24 h) and later (48 h vs. 7 days) time-points were also investigated to determine dynamic effects of injury within the first day and week after FPI. For western blot data analysis, the ratio of receptor volume to total protein volume was compared across groups. When *p* < 0.05 for the overall ANOVA was reached, Bonferroni corrected post hoc pairwise comparisons were performed. See Supplementary Figure S1 for representative blots in detected significant effects.

## Results

**FIG. 2.**
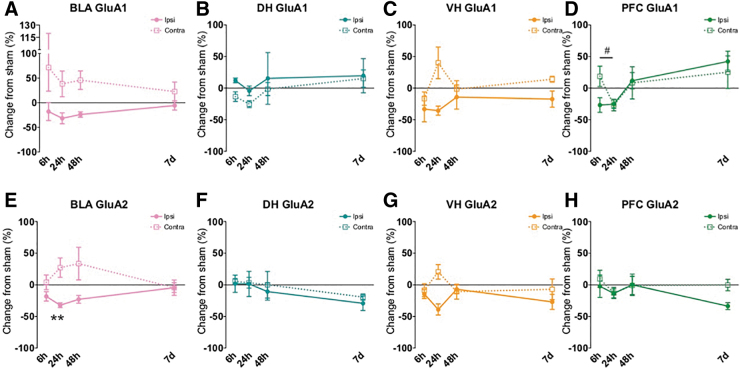
Synaptic a-amino-3-hydroxy5-methyl-4-isoxazolepropionic acid receptor (AMPAr) subunit changes early after lateral fluid percussion injury (FPI). There was a significant decrease of the GluA2 AMPAr subunit, in ipsilateral basolateral amygdala (BLA) 24 h after FPI compared with sham **(E)**, and a significant decrease in GluA1 in contralateral medial prefrontal cortex (PFC) across time within the first day after injury **(D)**, with no other significant changes in AMPArs. Data are represented as mean ± SEM (standard error of the mean); **p < 0.01, relative to sham within each blot; #*p* < 0.05 FPI 48 h vs. FPI 7 days.

**FIG. 3.**
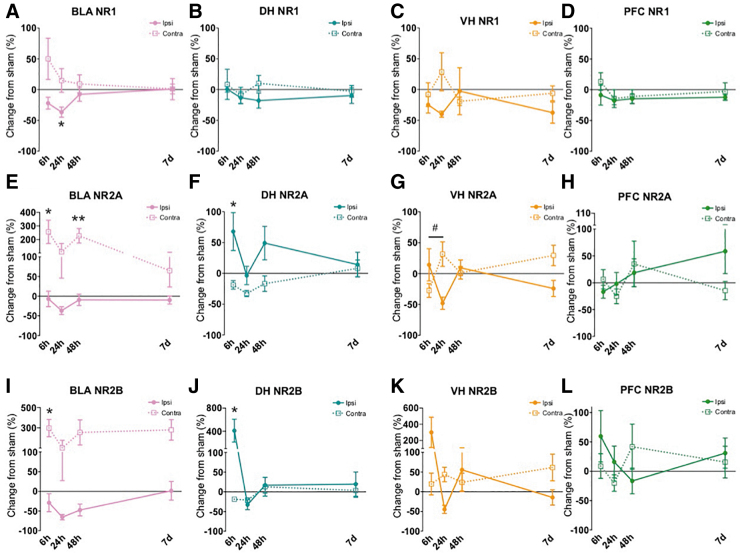
Synaptic *N*-methyl-D-aspartate (NMDA) receptor subunit changes early after lateral fluid percussion injury (FPI). In the ipsilateral basolateral amygdala (BLA), there was a significant decrease in NR1 24 h following injury **(A)**. We also saw significant elevations at 6 h in NR2A and NR2B in contralateral BLA **(E,I)**, where NR2A was still elevated in contralateral BLA 48 h after FPI (E). In the ipsilateral dorsal hippocampus (DH), both NR2A and NR2B were increased at 6 h after impact **(F,J)**. There was also a significant increase across time for NR2A in contralateral ventral hippocampus (VH) within the first day of injury. No other significant differences were observed for synaptic NMDArs. Data are represented as mean ± SEM (standard error of the mean); **p < 0.01, *p < 0.05, relative to sham within each blot; #*p* < 0.05 FPI 6 h vs. FPI 24 h in contralateral VH.

**FIG. 4.**
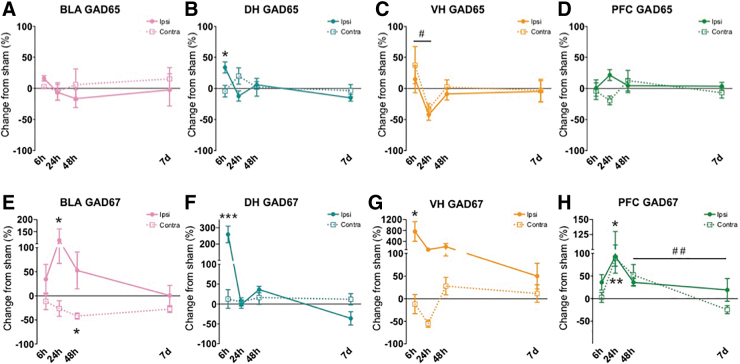
Alterations in gamma-aminobutyric acid (GABA) synthesis-related proteins early after lateral fluid percussion injury (FPI). In the ipsilateral basolateral amygdala (BLA), GAD67 was increased at 24 h and decreased in the contralateral BLA at 48 h after FPI **(E)**. Both GAD65 and GAD67 were significantly increased in the ipsilateral dorsal hippocampus (DH) 6 h after injury **(B,F)**. GAD67 was also increased in the ipsilateral ventral hippocampus (VH) 6 h after FPI, where GAD65 decreased from 6 h to 24 h following FPI. **(G)**. In the medial prefrontal cortex (PFC), GAD67 was increased bilaterally at 24 h following injury, and significantly decreased from 48 h to 7 days in contralateral PFC. **(H)**. Data are represented as mean ± SEM (standard error of the mean); ***p < 0.001, **p < 0.01, *p < 0.05, relative to sham; #*p* < 0.05, ##*p* < 0.01 across injury time-points within each blot.

Table 1 characterizes subjects and FPI for all groups. Using a one-way ANOVA, there were no significant differences in duration of apnea (F[3,24] = 0.726, *p* > 0.1), return of reflex (toe pinch withdrawal; F[3,24] = 0.538, *p* > 0.1), or atmospheres of pressure delivered to the dura (F[3,24] = 2.968, *p* > 0.05) across injury groups. Changes across groups are displayed in the figures and sorted by protein type (Fig. 2: AMPA receptor subunits, Fig. 3: NMDA receptor subunits, Fig. 4: GAD proteins). Rows depict different subunits/isoforms as indicated by graph titles. The colored columns are conserved across figures and portray brain region (BLA: pink, DH: teal, VH: yellow/orange, PFC: green). In the text, results are explained by brain region (correlates with graph color) and separated by excitatory-related (Figs. 2 and 3) and GAD proteins (Fig. 4).

### Basolateral amygdala (BLA)

#### Excitatory-related proteins

As depicted by the pink graphs in Figure 2 (A,E) and Figure 3 (A,E,I), in the ipsilateral BLA, a one-way ANOVA revealed a significant group effect for GluA2 (Fig. 2E) and NR1 (Fig. 3A) at early time-points (GluA2 for Sham, 6 h, 24 h: F[2,19] = 6.436, *p =* 0.008; NR1 for Sham, 6 h, 24 h: F[2,20] = 4.663, *p =* 0.023). Post hoc comparisons revealed a significant decrease in ipsilateral BLA GluA2 at 24 h relative to sham, *p =* 0.007 and for ipsilateral BLA NR1 at 24 h relative to sham, *p =* 0.022 (Fig. 2E).

In the contralateral BLA, a one-way ANOVA revealed a significant group effect for NR2A at both early and later time-points (NR2A for Sham, 6 , 24 : F[2,20] = 4.299, *p =* 0.01; NR2A for Sham, 48 h, 7 day: F[2,20] = 8.067, *p =* 0.003, Fig. 3E). There was also an effect of group at the early time-points for contra BLA NR2B (Sham, 6 h, 24 h: F[2,20] = 5.037, *p =* 0.018; Fig. 3I). Post hoc analyses revealed that both NR2A and NR2B were significantly increased at 6 h relative to Sham in contralateral BLA (NR2A, *p =* 0.027; NR2B, *p =* 0.018). NR2A was also increased at 48 h relative to Sham (*p =* 0.003). No other effects were significant for BLA excitatory proteins.

#### GAD proteins

As depicted by the pink graphs in Figure 4 (A,E), inhibitory-related protein GAD67 had a significant group effect in the ipsilateral BLA at early time-points (Sham, 6 h, 24 h: F[2,21] = 3.44, *p =* 0.05) and a significant group effect at later time-points in contralateral BLA (Sham, 48 h, 7 day: F[2,20] = 6.003, *p =* 0.01; Fig. 4E). Post hoc analyses revealed that GAD67 was increased at 24 h vs. Sham (*p =* 0.05) ipsilaterally, whereas contralaterally GAD67 was decreased at 48 h vs. Sham (*p =* 0.011). There were no significant changes for GAD65 in the BLA.

### Dorsal hippocampus (DH)

#### Excitatory-related proteins

As seen in the teal graphs in Figure 2 (B,F) and Figure 3 (B,F,J), in the ipsilateral DH, a one-way ANOVA showed a significant group effect for both NR2A and NR2B at 6 h post-injury (NR2A for Sham, 6 h, 24 h: F[2,18] = 6.111, *p =* 0.009; NR2B for Sham, 6 h, 24 h: F[2,17] = 5.389, *p =* 0.015; Fig. 3F,J). Post hoc tests revealed a significant increase in both ipsilateral DH NR2A and NR2B levels 6 h post-injury relative to sham, *p =* 0.021 and *p =* 0.032, respectively. No significant effects in any of the targeted excitatory proteins at any other time-points were found in the ipsilateral or contralateral DH.

#### GAD proteins

One-way ANOVA revealed significant differences in both GAD65 and GAD67 proteins in the ipsilateral DH 6 h post-injury as shown in the teal graphs of Figure 4 (B,F). (GAD65 for Sham, 6 h, 24 h: F[2,17] = 9.524, *p =* 0.002; GAD67 for Sham, 6 h, 24 h: F[2,19] = 30.568, *p* < 0.001; Fig. 4B,F). Post hoc comparisons showed both GAD65 and GAD67 were significantly increased at 6 h compared with sham, *p =* 0.017 and *p* < 0.001, respectively. At later time-points one-way ANOVA also showed changes in GAD67 in the ipsilateral DH (GAD67 for Sham, 48 h, 7 days: F[2,19] = 7.279, *p =* 0.004). Post hoc tests revealed a significant change in DH GAD67 between 48 h and 7 days, whereas GAD67 decreased significantly across time within the first week of FPI (*p =* 0.004). No other significant changes from sham were found at any time-point in either ipsilateral or contralateral DH for GABA-synthetic proteins.

### Ventral hippocampus (VH)

#### Excitatory-related proteins

All results are depicted by the yellow/orange graphs in Figure 2 (C,G) and Figure 3 (C,G,K). A one-way ANOVA revealed changes in NR2A in contralateral VH at early time-points (NR2A for Sham, 6 h, 24 h: F[2,19] = 3.949, *p =* 0.037). Post hoc comparisons revealed that within the first day of injury, in the contralateral VH, NR2A significantly increased between 6 and 24 h after FPI (*p =* 0.011; Fig. 3G). No other significant differences were found for the remaining excitatory proteins at any other time-point in the ipsilateral and contralateral VH.

#### GAD proteins

For GAD65 and GAD67, significant changes were observed in the ipsilateral VH at early time-points (GAD65 for Sham, 6 h, 24 h: F[2,18] = 4.033, *p =* 0.036; GAD67 for Sham, 6 h, 24 h: F[2,18] = 3.952, *p =* 0.038; Fig. 4C,G). Bonferroni corrected post hoc comparisons revealed that GAD67 was elevated compared with Sham in the ipsilateral VH at 6 h, *p =* 0.050. Post hoc comparisons of GAD65 protein in the ipsilateral VH at early time-points revealed a significant decrease in GAD65 between 6 h and 24 h after FPI, *p =* 0.039. No other significant GAD protein alterations were found at any other time-point in the ipsilateral or contralateral VH as shown in Figure 4, yellow/orange graphs (C,G).

### Medial prefrontal cortex (PFC)

#### Excitatory-related proteins

As depicted by the green graphs in Figure 2 (D,H) and Figure 3 (D,H,L), no significant differences were found in any excitatory-related proteins in the ipsilateral PFC. The only significant change of excitatory-related proteins in the PFC occurred in GluA1 in the contralateral hemisphere at earlier time-points (GluA1 for Sham, 6 h, 24 h: F[2,18] = 3.614, *p =* 0.048). Post hoc comparisons showed a significant decrease in contralateral PFC GluA1 across time within the first day of injury, (6 h vs. 24 h, *p =* 0.048). There were no differences in contralateral PFC for excitatory-supporting proteins.

#### GAD proteins

As shown in the green colored graphs in Figure 4 (D,H), GAD67 was found to be significantly altered in the PFC at early time-points (GAD67 Sham, 6 h, 24 h for ipsilateral PFC: F[2,19] = 3.904, *p =* 0.038). There were also significant differences in the contralateral PFC at both early and later time-points for GAD67, but not GAD65; (GAD67 for Sham, 6 h, 24 h: F[2,17] = 8.657, *p* = 0.003: GAD67 for Sham, 48 h, 7 days: F[2,16] = 6.500, *p =* 0.009; Fig. 4H). Post hoc Bonferroni correction comparisons revealed a significant bilateral increase in GAD67 at 24 h in the PFC versus shams; *p =* 0.036 (ipsilateral), *p =* 0.004 (contralateral), Figure 4H. We also saw a significant change across time within the first week of injury where contralateral PFC GAD67 was significantly decreased from 48 h to 7 days after FPI (*p =* 0.009).

## Discussion

This study investigated the impact of lateral FPI on the balance of neuroplasticity-related proteins in bilateral limbic structures including the amygdala, DH, VH, and medial PFC. We explored the subacute time course from 6 h to 1 week after lateral FPI for changes in proteins that support synaptic excitatory (synaptic NMDA and AMPA receptor subunits) and inhibitory processes (GAD65 and GAD67), with an *a priori* hypothesis that the BLA would exhibit increased excitatory-related and/or decreased inhibitory-related protein expression. Our results showed that NMDAr subunit NR1 and AMPAr subunit GluA2 were decreased in the ipsilateral BLA 24 h after injury. However, we also found that NR2A and NR2B were both increased in contralateral BLA, whereas GAD67 was decreased at 48 h post-FPI.

**FIG. 5.**
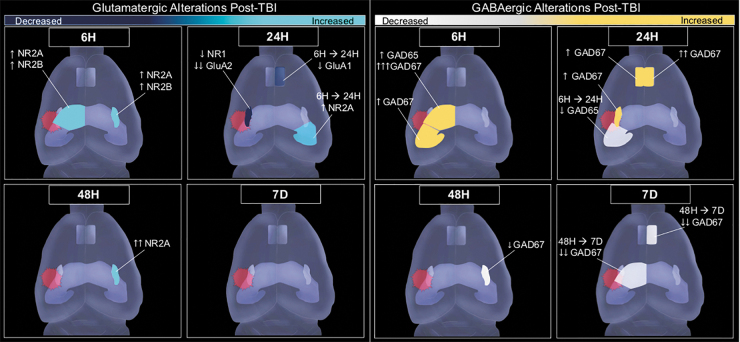
Summary of glutamatergic (left) and gamma-aminobutyric acid (GABA) synthesis-related proteins (right) changes after lateral fluid percussion injury (FPI). Number of arrows represent significance value (↑ / ↓ represents 0.01 < *p* < 0.05, ↑ ↑ / ↓ ↓ represents 0.001 < *p* < .01, ↑ ↑ ↑ / ↓ ↓ ↓ represents *p* < 0.001). Spiked red circle indicates site of injury.

Within the hippocampus, we saw synaptic changes limited to the ipsilateral hemisphere in the dorsal subregion (DH) with an increase in excitatory-related NR2B and a massive surge in GABA-synthetic proteins GAD65 and GAD67 acutely at 6 h after injury. Similarly, in the ipsilateral VH we also saw a robust increase of GAD67 6 h after impact. In the medial PFC, there was a bilateral increase of GAD67, and a decrease in GluA1 in contralateral PFC from 6 h to 24 h after injury. Then at later time-points, GAD67 was decreased in the contralateral PFC significantly from 48 h to 7 days post-injury, highlighting the dynamic change across time in response to lateral FPI. Collectively, our molecular findings reflect early changes proximal to the site of injury, and later to more distal areas (see Fig. 5). These results suggest a dynamic and complex response to injury across limbic networks that may represent a period in which these neural substrates that mediate emotion and cognition are perturbed.

### Excitatory/inhibitory balance after TBI

NMDAr and AMPAr excitatory-related proteins have known roles in both experience-dependent plasticity and disease.^39,40^ Optimal balance of excitatory receptor protein expression and function are necessary for adaptive synaptic transmission. Too little may result in impaired cognitive function and neurological deficits, whereas pathological glutamatergic activation via NMDAr and AMPAr may underlie increases in susceptibility to excitotoxic conditions such as post-traumatic epilepsy^41^ or even sensitized fear reactions.^42^ When considering the general patterns of excitatory/inhibitory response observed in our data, a pattern emerged where proteins that support excitatory plasticity tended to be decreased nearest to the site of injury and increased contralaterally. Conversely, the GAD proteins were increased ipsilaterally and decreased contralaterally. This pattern may reflect a homeostatic compensatory response following injury, promoting an effort to maintain optimal balance. Following biomechanical impact to the brain, there is an immediate (within minutes) increase in extracellular potassium and an indiscriminate wave of glutamate release in the ipsilateral DH.^43^

Within the ipsilateral DH and VH we found a robust increase of GAD67, an enzyme that converts glutamate to GABA. This surge in GABAergic synthesis within hours of lateral FPI may reflect regulatory mechanisms to restore excitatory/inhibitory balance in the acute phase given that these levels are relatively restored at later time-points. This apparent balance of responses between excitatory and inhibitory supporting proteins relative to the injury site supports the hypothesis of a molecular homeostatic response to brain injury. Many of the acute (within 24 h after injury) changes we observed stabilized to sham level over this short time course. However, some effects were persistent or only began to emerge a week following injury, such as in the PFC. Perhaps these changes reflect important targets for the longer course of a chronic disease process after TBI. An important limitation to acknowledge in our study is that our protocol did not account for cell-type specificity, which could occlude overall excitatory/inhibitory-related changes if they occurred primarily in a particular cell type. Future immunohistochemistry studies will complement our current findings and address these questions with cell type and microcircuit anatomical specificity.

### Plasticity changes in the amygdala

In the current study we observed dynamic changes in proteins that support excitatory and inhibitory processes relative to the site of injury and across limbic structures within the first week after lateral FPI. Given these varying effects of injury over time within the adult brain, it is important to consider these changes with respect to the dominant function of each structure. We hypothesized that plasticity within the amygdala would show an overall elevation by either increased glutamatergic and/or decreased GABAergic protein expression after FPI, reflecting elevated excitability. Although we observed a decrease in synaptic AMPAr (GluA2) ipsilaterally, we also observed increased NMDAr and reduced GABAergic proteins contralaterally. Interestingly, the GluA2 subunit renders the AMPAr Ca2+ impermeable, and is reduced following *in vitro* and *in vivo* injury models.^44,45^ Reductions in surface GluA2 following injury have been shown to increase expression of GluA2-lacking AMPArs, which increase Ca2+ permeability and elevate the susceptibility for secondary injury and cellular dysfunction.^44,45^

We previously showed increased Ca2+ permeable AMPArs in the BLA following our behavioral model of acute traumatic stress, and that this increase following stress is necessary for the expression of sensitized fear.^42^ Such changes in plasticity within the amygdala could contribute to a state of maladaptive stress reactivity, emotional learning, and psychiatric affect and mood comorbidities^22^; however, this idea in TBI remains to be further explored. A recent retrospective clinical study found increased amygdala volume in veterans with comorbid TBI/PTSD relative to those with TBI alone in the chronic phase after injury.^46^ Studies in PTSD patient populations suggest that the amygdala is hyperreactive to emotional stimuli,^47–49^ which has also been demonstrated in rodent models of stress and fear learning.^50,51^ Interestingly, dendritic hypertrophy in the BLA has been observed within 1 day and up to at least 28 days following midline FPI.^21^ Recent work from our lab showed that fear learning is increased early (48 h) after lateral FPI,^1,2,35^ which in the current study is when we observed increased excitatory-related and decreased GAD proteins in the contralateral BLA using this same model. Another study showed changes in BLA GAD67 with decreased GAD67 in both ipsilateral and contralateral BLA with corresponding increased anxiety-like behavior 1 week following lateral controlled cortical impact.^20^ Thus a net increase in plasticity within the BLA could, in part, represent a state of vulnerability for increased emotional learning and stress reactivity after TBI.^22^

In addition to increased sensitivity to stress after TBI, increased amygdala excitability after physical brain insult is also linked to post-traumatic and temporal lobe epilepsy.^52^ The current study did not investigate behavior or other functional outcomes; however, we speculate that these data further support a possible neurochemical mechanism behind such changes that has been previously observed in our model.^12,35^ It is important to acknowledge work with findings in contrast to our current and previous findings in the amygdala after TBI. For instance, Palmer and colleagues showed decreased BLA excitability and activation that corresponded with a significant decrease in freezing behavior after cued fear conditioning.^53^ Another study found decreased evoked glutamate release and slower glutamate clearance in the central amygdala after midline FPI in rats.^54^ These studies were completed using slightly different models and time courses than the current study (mice, 7–8 days post right parietal FPI^53^; 7 and 28 days post midline FPI in a proximal amygdala subregion^54^). However, together this work demonstrates that excitatory- and inhibitory-related mechanisms in amygdala networks are vulnerable to disruption after TBI and offers an important target for future research in the scope of heterogeneous emotional comorbidities in TBI.

### Plasticity changes in the hippocampus and prefrontal cortex

Similar to the BLA, the hippocampus and PFC are involved in the processing of fear memory,^55–57^ and are common foci for neuropathology and functional changes following TBI. The hippocampus mediates the encoding of contextual and declarative memory in general,^58–61^ and also provides negative feedback regulation of the hypothalamus-pituitary-adrenal stress response.^62^ The lateralized effects of FPI on the hippocampus were found to be variable over time and included dynamic changes in synaptic proteins. These fluctuations could be factors underlying behavioral changes after injury. Although studies that investigate hippocampal changes after TBI tend to consider the whole structure, or keep focus to the dorsal subregion, it is important to highlight that the DH and VH hold functional and connective distinctions that mediate cognitive and emotional function differently.^27^ To our knowledge, our study is the first to examine the functional differences between the DH and VH within subject following lateral FPI.

Although we observed a similar increase of GAD67 at 6 h after impact in both ipsilateral DH and VH, we did observe unique differences between the two subregions. Synaptic NR2A, NR2B, and GAD65 were all significantly increased 6 h after FPI in ipsilateral DH, whereas these changes were not observed in the VH. However, NR2A was increased in the contralateral VH across time in the first day (6 h vs. 24 h). Past work from our lab on NR2A and NR2B changes following FPI did not see significant changes in ipsi- or contralateral whole hippocampus (DH and VH combined) from 1 to 14 days post-injury.^11^ Although the previous study did not investigate as early as 6 h after injury, when we saw NR2A and NR2B changes, the findings are consistent with our current data from 24 h and 48 h. Our findings add to the growing body of literature supporting other functional plasticity regulators that are also elevated within 1 week after FPI including hippocampal phosphorylated cAMP-response element binding protein (pCREB) and (p)synapsin I.^63^ Reduced cortical NMDAr binding^64^ as well as impaired hippocampal long term potentiation (LTP) induction have also been observed in other studies of preclinical TBI.^65,66^ We also observed changes in the medial PFC, with initial increases in GAD67 bilaterally at 24 h and a contralateral decrease in GluA1. Further, there was a significant decrease in GAD67 in contralateral PFC from 48 h to 7 days post-injury. This lasting effect of TBI in the PFC could reflect a functional deficit and/or PFC network disruption.

Another study using lateral FPI showed reduced spine density in medial PFC neurons and impaired fear extinction weeks after injury.^67^ Another recent study using proton magnetic resonance spectroscopy after midline mild TBI in mice showed increased GABA levels 8 days after injury, as well as increased fear learning and impaired fear extinction.^68^ Although our data revealed an earlier increase of GABA synthesis (GAD67) at 24 h after FPI and a subsequent decrease at 7 days contralaterally, it is interesting to note that both injury models support early elevated GABAergic mechanisms and corresponding increases in fear learning^12,35,68^; however, they did not observe significant changes in the amygdala. Clinically, PFC dysfunction after TBI can be long lasting and often manifests in problems with inhibitory control,^69–71^ and other behavioral sequelae related to emotion regulation.^72^ Balanced activity and function within the interconnected network of the amygdala, hippocampus, and PFC is essential for optimal cognition, adaptive stress reactions, and emotional regulation. Our study and others have demonstrated these networks to be vulnerable to TBI leading to altered network plasticity.

An important limitation to consider in our study is that although we differentiated between DH and VH, there are also multiple subregions within the hippocampus (i.e., dentate gyrus, CA1, CA3), that serve differential roles in excitation and inhibition^73^ and have important implications for effects post-TBI.^74,75^ Further, the PFC section that we collected includes subregions as well (anterior cingulate, prelimbic, and infralimbic cortices) that are interconnected and have been shown to serve differential roles in stress and emotion regulation.^76,77^ The findings we report therefore reflect a net effect across these subregions, where future work will focus on differential analysis in and across such microcircuits within regions.

## Conclusion

The current study evaluated lateralized effects of FPI across interconnected limbic structures and the medial PFC within the first week of injury. We observed a dynamic, lateralized homeostatic response to lateral FPI that generally resolved by 1 week after insult under these conditions. However, the early disruptions in molecular signatures that control synaptic plasticity and inhibitory tone across systems that regulate emotion and cognition may reflect a vulnerable window for elevated stress sensitivity and subsequent development of psychiatric comorbidities. This translational research provides insight as to how the observed neuromolecular changes in male rodents after TBI could affect function and thus may be contributing to the elevated comorbidity of emotional disorders and other dysfunction after injury that we see clinically.

## Supplementary Material

Supplemental data

## Abbreviations Used

AMPA: a-amino-3-hydroxy5-methyl-4-isoxazolepropionic acid
AMPArs: AMPA receptors
ANOVA: analysis of variance
BLA: basolateral amygdala
CH: crude homogenate
DH: dorsal hippocampus
FPI: fluid percussion injury
GABA: gamma-aminobutyric acid
GAD: glutamate decarboxylase
LOC: loss of consciousness
LTP: long term potentiation
NBA: National Basketball Association
NCAA: National Collegiate Athletic Association
NFL: National Football League
NHLPA: National Hockey League Players' Association
NMDA: *N*-methyl-D-aspartate
NMDArs: NMDA receptors
pCREB: phosphorylated cAMP-response element binding protein
PFC: prefrontal cortex
PTSD: post-traumatic stress disorder
SEM: standard error of the mean
SNS: synaptic fractions
TBI: traumatic brain injury
TBST: Tris-buffer saline with Tween 20
UCLA: University of California, Los Angeles
VH: ventral hippocampus
