# Supplementary figures and images for: Region-Dependent Modulation of Neural Plasticity in Limbic Structures Early after Traumatic Brain Injury
Source: Neurotrauma Rep. 2021 Apr 8;2(1):200–13. doi: 10.1089/neur.2020.0045 (PMC8086520; doi:10.1089/neur.2020.0045)

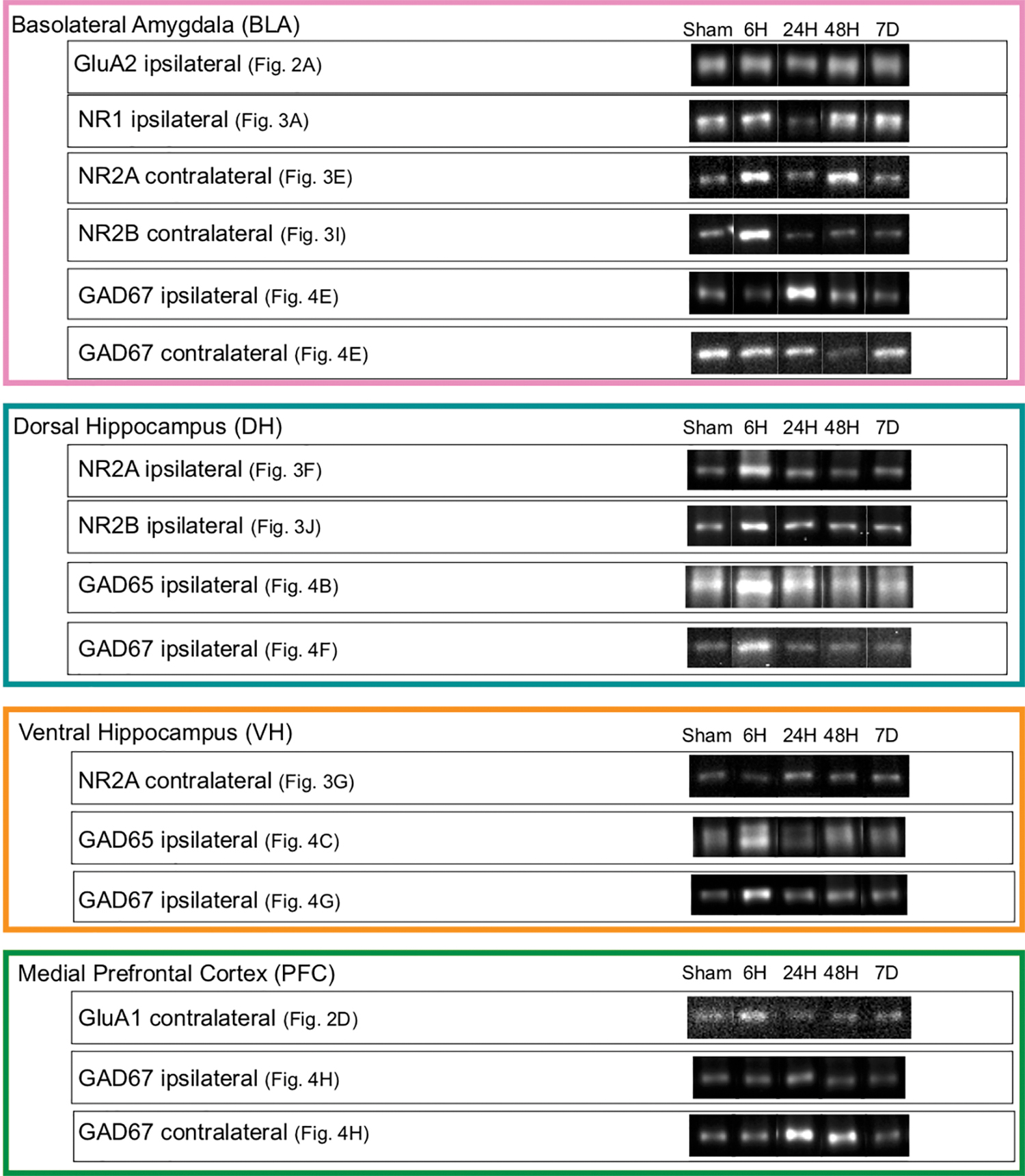

Supplement: Supplemental data [file Supp_FigureS1.jpg]
